# Hattangadi Shashidhar Bhat - “Guru”

**DOI:** 10.4103/0970-1591.44244

**Published:** 2008

**Authors:** Ganesh Gopalakrishnan

**Affiliations:** Department of Urology, Christian Medical College, Vellore - 632 004, Tamil Nadu, India

**Figure:**
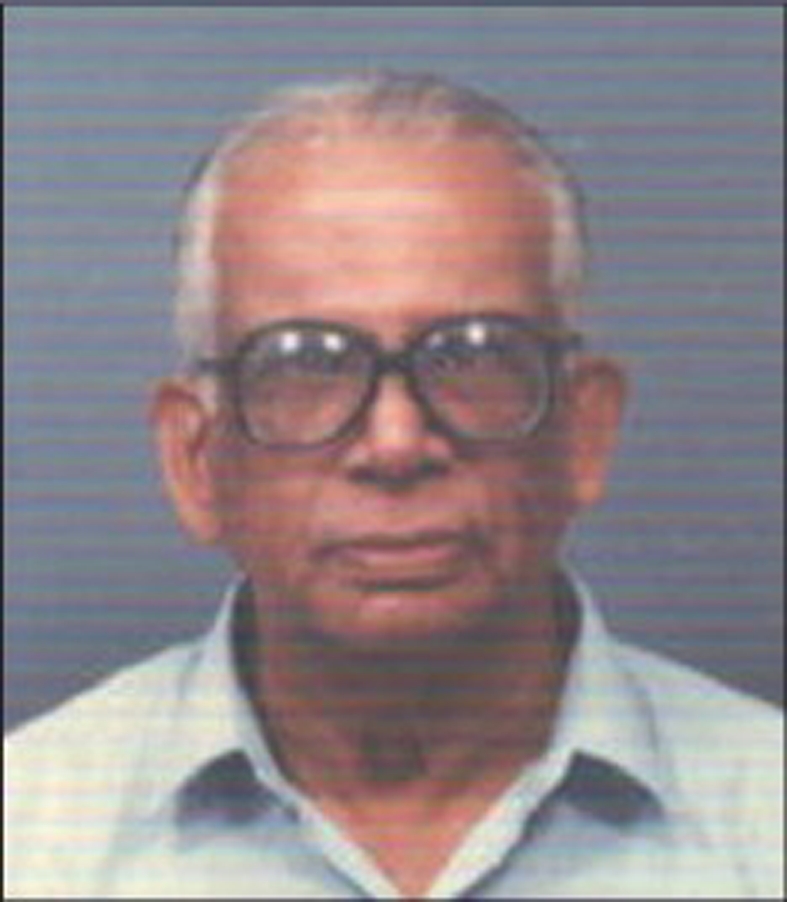
Dr. Hattangadi Shashidhar Bhat

Hattangadi Shashidhar Bhat, HSB as he is fondly known to all of us was born into a simple family in Udipi on 21 January 1921.

After his early school education in Mangalore he went on to complete his undergraduate medical studies from Stanley Medical College, Chennai. He was the best student in his batch and showed a keen interest in surgery in his early days. One might call it coincidence or Divine design that Dr. Robert Cochrane, the Director of Christian Medical College (CMC), Vellore called HSB to join the institution.

After HSB joined CMC in 1945 he was credited as being the most wanted house officer. He worked in departments of Oto-Rhino-Laryngology, Obstetrics and Gynecology, Casualty, General Surgery and also did a stint in neurosurgery. He completed his Masters in Surgery (MS) in General Surgery in 1953.

How does one do justice to a festschrift, to a man fondly described as the doyen of Indian Urology, the teacher of teachers? How does one bring out his spirituality in the practice of medicine and surgery?

I would like to focus on HSB the man, HSB the urologist and teacher and HSB the family man.

## HSB THE MAN

‘To be very honest, God has given me more than I deserve, considering my academic background which should have relegated me to a general practitioner only. This makes me firmly believe that to be a successful consultant a few essentials are needed in a medical professional. These include God's grace, an accurate understanding of the fundamentals and basic principles of surgical techniques and intense desire to do unto the patient what you would like done unto yourself and expectation of nothing in return for your services”.

HSB is a no-nonsense man, a stickler for detail and downright honest who will call “a spade a spade”. Whenever he conducted an inspection for a department or facility he would inform the authorities that all he needed was a closed room, a table and chair to write his report with “no interference” from anyone. In short, he did not want any favors nor to be obliged to anyone.

Patients usually come to an institution for its quality care. Patients came to CMC for HSB. He was compassion personified. He firmly believed that there is no such thing as “cheap treatment, only correct and low-cost treatment”. Illness is the birthright of every human irrespective of his or her economic or social status. “What is the difference between a beggar and an emperor?” asks HSB rhetorically.

## HSB THE SURGEON AND TEACHER

“Equanimity is a quality we surgeons must develop, because some patients may deteriorate despite the best care and surgery and healing is not entirely in our hands” he says. In fact he candidly admits that even though he is a surgeon his first thought is always whether a person can be treated without surgery and regards it as indicative of a surgeon's maturity. He believes surgery is an art that relies on scientific methods for correct diagnosis and treatment. Practice in the art of healing does not always follow textbook rules nor is it entirely dependent on scientific calculation. This is because the working of a human body is not yet an open book, in spite of all the advances of science”.

There were four mentors in the surgical school of HSB. Dr. Somerwell, Prof. Raghavachari, Prof. John Spencer Carman and Prof. Roger Barnes. Each one of these individuals shaped his life and his “take on life”.

HSB himself was a very skilled surgeon and his skills were legendary. His surgical mentor Dr. Somerwell was so sure of HSB's abilities that he chose him to operate on his hernia and that too under local. Dr. Somerwell insisted that HSB use the same technique he taught him and wanted him to show him the ilioinguinal nerve before dividing it. Dr. Somerwell supervised the operation reclining on three pillows during the entire procedure.

Prof. Raghavachari was a clinician “non-pareil” and helped HSB in his surgical teaching. They would hold classes on Sundays and the only time classes were cancelled was when there was a cricket match as Prof. Raghavachari was a cricket enthusiast!

Prof. Carman was the chief of surgery in Vellore from 1954-67 and was a great influence on HSB in his formative years. Dr. Carman kindled the urological interest in HSB and after working for 10 years under him, HSB was given charge of the unit and to develop urology. Carman himself had no formal urological residency training in the US but had worked with a urologist for a year on a furlough.

If anyone has not read HSB's MS thesis then ask him to loan it to you. It is like a urological encyclopedia. Numerous cases are detailed accurately, why certain decisions on management were taken, what was the outcome of treatment of the patient and the current references on the subject.

It was Roger Barnes from Loma Linda University in California who was instrumental in bringing the art of Transurethral resection of prostate (TURP) to India in 1956-57. HSB claims that he was baptized by Roger Barnes in the art of TURP. It was in the department library that Dr. Barnes authored the ninth volume of the Encyclopedia Urologica section of Endoscopic Urologic Surgery. The illustration for this book was drawn by Mr. Giri, CMC's hospital artist. Prof. Barnes dedicated the book to the Urologists of India who are struggling to establish urology as a specialty.

HSB says “These four teachers of surgery molded me with their behavior towards patients and relatives, their concern for their assistants, interaction with colleagues and their concern for trainees. Great teachers are not those who dole out facts from a textbook but who pass on their enthusiasm to those assigned to them for training and help them to develop further on their ideas”.

In 1965, the Vice-Chancellor of the Madras University was Dr. A Lakshmanaswamy Mudaliar. It was Roger Barnes who did the TURP on Dr. AL Mudaliar and the story goes that this paved the way for starting the MCh course in Urology in Madras University - simultaneously in Christian Medical College, Vellore and Government General Hospital (GH) and Madras Medical College, Chennai where Dr. AL Mudaliar's son Dr. A Venugopal was the Chief of Urology.

It is to HSB's credit that he took on the mantle of starting a department, nurturing it and made it grow. The growth spread outwards as Chiefs of Surgery from other institutions came to CMC to train under HSB in Urology. All of them went back to establish departments in their respective states, medical colleges and the Indian Army. And to his credit, he organized the first Urological Society of India (USI) meet in Vellore in 1961.

To his patients HSB was God. The ward was the extension of his home. Whenever he felt uncomfortable about the progress of patients he would go immediately and see them day or night! It was difficult for his residents to keep pace with him or his whereabouts. While the trainees would be waiting in one ward for rounds they would find that he had come much earlier, done the rounds, the dressings, written the order and gone back to the office to handle his administrative responsibilities.

HSB's teaching was not didactic. It was inspirational and philosophical. As a houseman myself I would see the entire Department of Urology every Sunday in the hospital canteen between 9 am-12 noon discussing urological philosophy, politics and other mundane matters.

## HSB THE FAMILY MAN

HSB and Prema are blessed with four lovely children. All of them are chips off the old block - fiercely independent, confident and with a keen sense of focus on work and life. Usha works in CMC as a clinical pathologist and is respected and acknowledged for her ability to discern subtle abnormalities in the blood. Tara is a microbiologist and took after her mother and currently is in charge of this service in Frontier Life Line Hospital in Chennai. Gurudutt is the odd man out and is an engineer who works in Toronto. Sanjay has taken after his father. He is a urologist who trained in CMC Vellore and now runs the department at Amrita Institute of Medical Sciences in Kochi. He is knowledgeable, extremely competent and a thoroughly honest individual who knows his limitations.

HSB is the perfect family man - husband and father. In 1945 his late wife Prema was involved in an accident while returning from a picnic. She insisted that only HSB should take care of her.

She was from Calicut and had heard that there was this handsome young surgeon from Udipi who was still unmarried. The rest is a sublime love story. Prema Bhat became a renowned microbiologist and did pioneering work on urinary tuberculosis for the Indian Council of Medical Research (ICMR). Both HSB and Prema Bhat lived and breathed CMC - not only for the three decades they lived here but also after leaving the institution. This is what they have to say “The training and service at CMC for nearly three decades was a continuous learning process. It taught us what a complete hospital should be and what caring for the sick and ailing was all about. Internationally staffed and with Indians belonging to different regions, religions and philosophies, we were all proud of the label made- in- CMCH”. He opted for premature retirement from CMC in 1975.

HSB is a God-fearing man. He is currently the Chairman of the Uro-Nephrology Division, Sri Sathya Sai Institute of Higher Medical Sciences (SSSIHMS), Puttaparthy. An ardent devotee of Sri Sathya Sai Baba, at 87 years, HSB is living in the abode of Sri Sathya Sai Baba at Puttaporthy. As he says currently he gets work done out of others, protects patients' prostates from trainee urologists, protects instruments from nurses and acts as a referee during boxing matches between urological colleagues, all this is by holding on to the tail of urology!

“There can be no practice of medicine and surgery without spirituality. As doctors we are HIS instruments. I firmly believe and fully realize that I can only cut but it is God who heals. Patients heal their disease in dialogue with God on a hotline” avers HSB.

As I pen these lines I have but one regret; I cannot credit myself as having being trained under HSB. He had just left CMC when I joined Urology. But I have no regrets. For in these years of being closely associated with him I have imbibed through listening and talking to him, facts of urology and life. I have learnt much more, heard much more and seen so much more of urology, life and humanity.

